# Cell Cycle Regulation of Stem Cells by MicroRNAs

**DOI:** 10.1007/s12015-018-9808-y

**Published:** 2018-03-14

**Authors:** Michelle M. J. Mens, Mohsen Ghanbari

**Affiliations:** 1 Department of Epidemiology, Erasmus University Medical Center, P.O. Box 2040, 3000 CA Rotterdam, The Netherlands; 2 Department of Genetics, School of Medicine, Mashhad University of Medical Sciences, Mashhad, Iran

**Keywords:** MicroRNA, Cell cycle, Stem cells, ESC, Somatic stem cell, Cancer stem cell

## Abstract

MicroRNAs (miRNAs) are a class of small non-coding RNA molecules involved in the regulation of gene expression. They are involved in the fine-tuning of fundamental biological processes such as proliferation, differentiation, survival and apoptosis in many cell types. Emerging evidence suggests that miRNAs regulate critical pathways involved in stem cell function. Several miRNAs have been suggested to target transcripts that directly or indirectly coordinate the cell cycle progression of stem cells. Moreover, previous studies have shown that altered expression levels of miRNAs can contribute to pathological conditions, such as cancer, due to the loss of cell cycle regulation. However, the precise mechanism underlying miRNA-mediated regulation of cell cycle in stem cells is still incompletely understood. In this review, we discuss current knowledge of miRNAs regulatory role in cell cycle progression of stem cells. We describe how specific miRNAs may control cell cycle associated molecules and checkpoints in embryonic, somatic and cancer stem cells. We further outline how these miRNAs could be regulated to influence cell cycle progression in stem cells as a potential clinical application.

## Introduction

### Stem Cells and Cell Cycle Regulation

Stem cells are characterized by their unlimited ability to self-renew and capability to differentiate into multiple cell lineages [1]. In this end, stem cells undergo an asymmetric cell division during which only one of the two daughter cells differentiates. This is a complex mechanism in which different transcription factors, epigenetic modifications and hormones are involved. There are two broad types of stem cells including embryonic stem cells (ESCs), which are solely present at the earliest stages of development, and somatic (or adult) stem cells, which appear during fetal development and remain throughout life. ESCs are pluripotent and therefore have the capacity to differentiate into all the possible cell types of the three germ layers. Somatic stem cells, however, are multipotent and can only differentiate into cell types of the specific tissue or organ from which they originate. It is also suggested that a certain type of stem-like cells is responsible for the initiation of cancer, so-called cancer stem cells (CSCs). It is thought that CSCs arise from either differentiated cancer cells or somatic stem cells [2].

In eukaryotes, the cell division cycle includes four discrete phases: Gap 1 (G1), Synthesis (S), Gap 2 (G2) and Mitosis (M). During the G1 phase, which is known as the first interphase, the cell synthesizes proteins that are needed for DNA replication and continuous growth. DNA replication takes place during the S phase and is followed by the G2 phase, which is known as the second interphase, where the DNA integrity is checked. At this point, the cell is growing and preparing for cell division. During the M phase, the cell divides into two daughter cells. After the mitotic phase, the daughter cells re-enter the G1 phase or go into the quiescent state. This is defined as a state of reversible cell cycle arrest and is known as the G0 phase [3]. The quiescent state is important for cellular homeostasis, meaning that it has the ability to either stop proliferating or to re-enter the cell cycle and self-renew when needed [4, 5].

The duration of the cell cycle and the transition from one phase to the next is highly variable between different cell types. While the cell cycle duration in murine somatic cells is relatively long (> 16 h), the duration in murine ESCs (mESCs) is much faster (8–10 h). A reduced G1 phase and prolonged S phase in ESCs are the causes that make this difference. In addition, human ESCs (hESCs) spend only 3 h in the G1 phase, compared to human somatic cells that spend 10 h in this phase [6]. The difference in cell cycle duration between ESCs and somatic stem cells is remarkable, an explanation could be that somatic stem cells are predominantly in a quiescent state compared to the fast dividing ESCs. Previous studies have indicated that the G1 phase is the most variable phase and that its duration contributes to cell fate determination [7–9].

When a cell enters the G1 phase, a protein called cyclin D increases in response to mitogenic stimuli. Cyclin D proteins bind to enzymes called CDK4/6 and together they form heterodimers. These complexes subsequently phosphorylate proteins of the retinoblastoma (*RB*) family. The *E2F* family is a group of genes encoding for transcription factors *E2F-1, E2F-2* and *E2F-3*, which are downstream targets of the *RB* family. The central member of the *RB* family, the *RB* tumor suppressor protein (pRb), is a negative regulator of the *E2F* genes. When pRb is hypophosphorylated, it inactivates *E2F* transcription factors, which results in the inhibition of transition from G1 to S phase. Hyperphosphorylation of pRb leads to dissociation of *E2F* from the E2F/pRb complex and contributes to the G1/S transition. Recent findings show the importance of the E2F/pRb activity in relation to ESCs self-renewal and differentiation [10–12].

Cyclin dependent kinase proteins (CDK) tightly regulate the progression of the cell cycle. A CDK binds to its regulatory cyclin protein partner to control the different cell cycle phases. Progression through S phase is regulated by the cyclin E-CDK2 complex, while the G2/M transition is under control of cyclin B-CDK1 complex. Cyclin dependent kinase inhibitor (CDKI) proteins including p21/Cip1, p27/Kip1 and p57/Kip2, block the activity of cyclin E-CDK2 and cyclin A-CDK1 [13]. Furthermore, proteins of the *INK4* family, including p16/INK4A, p15/INK4B, p18/INK4C and p19/INK4D inhibit the cyclin D-CDK4/6 activity. These mechanisms can lead to cell cycle arrest and are of major importance to regulate tissue homeostasis and prevent tumorigenesis. The p53-p21 signaling pathway is also involved in the transition of G1 to S phase and G2 to M phase. It is well established that loss of p53 is the main reason for genomic instability as the p53-null cells have disrupted the G1/S checkpoint [14–17]. In addition, the expression levels of p53 and p21 in ESCs are important for the maintenance of pluripotency [18].

### Biogenesis of MicroRNAs

Epigenetic features, such as the activity of microRNAs (miRNAs), modulate the expression of cell cycle-associated genes [19–23]. MiRNAs are a conserved class of endogenously expressed small non-coding RNAs (spanning 20–24 nucleotides), that have been widely implicated in fine-tuning various biological processes. Since the discovery of the first miRNA in 1993 [24], the knowledge on miRNAs has been rapidly increased. MiRNAs are ubiquitously expressed in plants, animals and viruses, indicating the evolutionary importance of these small molecules. According to the miRBase database (v.21), 1881 miRNAs have been identified with confidence in human [25]. These miRNAs are suggested to regulate the expression of more than 60% of all protein-coding genes. Previous research has investigated the functional role of miRNAs in diverse mechanisms including cell proliferation, apoptosis, and differentiation. Additionally, alteration in the expression of miRNAs contribute to human diseases such as cancer and cardiovascular disease [26–33].

MiRNA maturation is a complex biological process that is subjected to tight molecular regulation. In the nucleus, miRNAs are initially transcribed as 800-3000nt long primary transcripts (pri-miRNA). These pri-miRNAs are subsequently cleaved by Drosha, RNaseII, endonuclease III, and Pasha/DGCR8 proteins to generate ~ 70nt hairpin precursor miRNAs (pre-miRNAs). Following this initial process, pre-miRNAs are transported to the cytoplasm by Exportin 5. Subsequently, the hairpin precursor is cleaved in a ~ 22nt double-stranded miRNA by the ribonuclease III enzyme called Dicer together with TRBP/ PACT proteins. The guide strand (5′ end) then associates with members of the Argonaute family and is been incorporated into the RNA-induced silencing complex (RISC). The miR-RISC complex facilitates base-pairing interaction between miRNA and the 3′ untranslated region (3′UTR) of target mRNA. The core of a mature miRNA, called the ‘seed’ region, includes nucleotides 2–7/8 from the 5′ end of the miRNA and plays a critical role in target recognition and interaction. Binding of the miRNA seed region to its complementary site in the target mRNA leads to translational repression or degradation of the target transcript.

The first studies investigating miRNA function in cell cycle regulation were published two decades ago, where two independent studies revealed that miRNAs lin-4 and let-7 induce cell cycle arrest in the nematode, *C. elegans* [24, 34]. Since then, several studies have demonstrated the importance of miRNAs in cell cycle regulation in different cell types including stem cells [21, 35, 36]. The role of miRNAs in stem cell proliferation was initially observed in knockout mice lacking Dicer and Dgcr8, which are key components of the miRNA biogenesis [37]. Dicer knockout mice were embryonic lethal and ESCs from Dicer-deficient mice exhibited defects in cell cycle progression [38]. Similarly, ESCs derived from Dgcr8-deficient mice exhibited delay in the cell cycle progression due to downregulation of genes involved in regulation of self-renewal [37]. These initial studies indicated that miRNAs are crucial for cell cycle regulation of stem cells. Then, other studies demonstrated that miRNAs are involved in the cell cycle progression of stem cells by direct or indirect targeting of different cell cycle-associated genes (e.g. Cyclins, CDKs and CDKIs). Understanding the tightly regulated networks of cell cycle in which miRNAs are interacting, will enhance our knowledge in the development of both healthy and disease states of the human body. In the following, we will discuss the recent advances on the functions of miRNAs in cell cycle regulation of stem cells. In addition, a promising therapeutic potential of miRNAs in controlling somatic and cancer stem cells self-renewal and proliferation will be discussed.

## MiRNAs and Cell Cycle Regulation of Stem Cells

### Embryonic Stem Cells (ESCs)

The duration of the cell cycle is variable between different types of stem cells. ESCs have a shorter cell cycle compared to somatic stem cells, which is due to a significantly abbreviated G1 phase and a prolonged S phase [39–41]. Previous studies have explored the phosphorylation status of pRb as a regulator for the length of G1 phase. Since mESCs lack cyclin D-CDK4 as well as cyclin E-CDK2, pRb will not be phosphorylated and thereby not stimulating the cyclin E-CDK2 activity [42]. Therefore, the time spent in G1 phase compared to S phase may be a key feature of the pluripotency fate [12]. Moreover, DNA damage response pathways, which are activated in the G1 phase, are reduced or absent in both hESCs and mESCs [43]. Several negative regulators of cell cycle progression, including p53, p16/INK4A, p19/ARF and p21/Cip1, are expressed at low levels in ESCs, while DNA repair and replication regulators are expressed at high levels [6, 43].

**Fig. 1:**
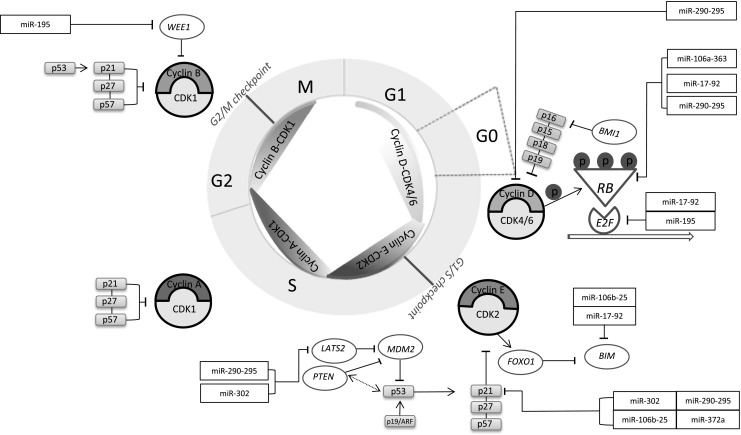
**An overview of cell cycle regulation in ESCs by miRNAs**. The figure illustrates the cell cycle progression in embryonic stem cells (ESCs). As shown, multiple key regulatory elements including cyclins, CDKs and CDK inhibitors are forming a network that progress cells through the four different phases of cell cycle. Several miRNA clusters and single miRNAs are involved in the regulation of cell cycle in ESCs by directly or indirectly targeting the cell cycle-associated components (e.g. *RB*, p53, p21, *LATS2, PTEN*, cyclin D, cyclin E). Among them, miR-17-92, miR-290-295, miR-302, miR-106b-25 and miR-106a-363 are abundantly expressed in ESCs. Inhibition of *E2F* by miR-92 and miR-195 decreases transcription of multiple transcription factors and proteins (e.g. *E2F-1, E2F-2, E2F-3, CDK2, CDC25A*), resulting in a reduction of G1 phase duration. Furthermore, the expression of main G1/S and G2/M checkpoint regulator p53 is decreased via indirect targeting by miR-290-295 and miR-302 in ESCs. This facilitates the G1/S transition. Moreover, p21 expression is reduced via miR-290-295, miR-372a, miR-302 and miR-106b-25 in a direct manner. This inhibits cyclin E-CDK2 activity, and therefore facilitates the G1/S transition. Additionally, miR-106b-25 and miR-17-92 can target pro-apoptotic gene *BIM*, resulting in a reduction of cells entering apoptosis [51]

Previous studies have shown the distinct expression pattern of miRNAs in ESCs. These studies demonstrate that ESCs express a set of miRNAs, of which a few are abundantly expressed at 60,000 or more copies per cell. The most abundantly expressed miRNAs in ESCs are miR-290-295, miR-302, miR-17-92, miR-106b-25 and miR-106a-363 clusters, which provide approximately 70% of the total miRNA molecules in ESCs [20, 44–46]. These miRNAs are expressed in homologous clusters, so-called polycistronic loci, which contribute to the same cis-regulatory elements [47]. The miR-290-295 cluster and miR-302 share a highly conserved seed-sequence ‘AAGUGCU’, while miR-17-92, miR-106b-25 and miR-106a-363 clusters share the seed-sequence ‘AAAGUGC’ [20]. These miRNAs are called the regulators of the embryonic stem cell cycle (ESCC), because of the ability in rescuing cell cycle progression in Dgcr8 knockout ESCs [20, 44, 48–50]. A schematic overview of the functionality of ESCC miRNAs is illustrated in Fig. 1. In general, ESCC miRNAs facilitate the G1/S transition mainly through suppressing the expression of RB proteins [44]. In addition, these miRNAs have been demonstrated to directly regulate the expression of p21/Cip1 and cyclin E-CDK2 regulatory molecules in mESCs, including *RB, RBL1, RBL2*, and *LATS2* [21, 48–50].

The miR-290 cluster, consisting of miR-291a-3p, miR-291b-3p, miR-294, and miR-295, is upregulated in undifferentiated ESCs, but is rapidly downregulated during differentiation [21, 50, 52]. It has been shown that members of this miRNA cluster promote the G1/S transition. Cells can relatively quick enter the S phase, because members of the miR-290-295 cluster directly target cyclin D-CDK4/6 and indirectly downregulate the cyclin E-CDK2 complex (Fig. 1). MiR-290-295 downregulates diverse inhibitors of the cell cycle, including *RB, RBL1, RBL2*, p21 and *LATS2*,which change the distribution of ESC in each cell cycle phase [47]. Furthermore, the miR-290-295 cluster enhances the somatic reprogramming by increasing the expression of pluripotent transcription factors *OCT4, SOX2, KLF4, LIN28, MYC* and *NANOG* [47, 53]. Also, miR-290-295 is shown to be directly involved to suppress apoptosis by targeting *Caspase 2* [54]. This leads to a reduced percentage of ESCs in G1 phase and an increased fraction of cells in S or G2/M phases. Due to the enhanced proliferation, the metabolism of ESCs rather rely on glycolysis than aerobic respiration. This metabolism is similar to the Warburg effect that is known in cancer cells [44, 47, 48]. Therefore, glycolysis-associated genes, such as *MYC, LIN28* and *HIF1*, have been promoted by the miR-290-295 cluster [44, 47]. Moreover, members of this miRNA cluster could affect epigenetic pathways including DNA methylation, histone acetylation and activation of Polycomb proteins, which inactivates genes involved in differentiation [55, 56].

The miR-17-92 cluster consists of miR-17, miR-18a, miR-19a, miR-19b, miR-20a and miR-92a. This miRNA cluster is crucial in early mammalian development by supporting cellular reprogramming and tumorigenesis [44]. In particular, miR-17-92 is a regulator of the *MYC* oncogene [51, 57]. *MYC* inhibits the expression of chromatin regulatory genes including *SIN3B, HBP1*, and *BTG1*, via miR-17-92. Through epigenetic mechanisms including reduced recruitment of histone deacetylase (*HADC)* via *HBP1*, miR-17-92 controls the chromatin stage of cell cycle related genes (Fig. 1) [51]. *MYC* through miR-17-92, contributes to the euchromatin formation of specific gene expression involved in DNA replication and repair mechanisms that goes along with a shift in the percentage of cells in a proliferating state [51]. Likewise, miR-106b, which shares a high sequence homology with miR-17 and miR-20a, is shown to promote G1/S transition by directly targeting p21, which results in a higher portion of cells in S phase compared to G1 phase [58].

The miR-302-367 cluster, consisting of miR-302a, b, c, d, and miR-367, has also been shown to play a crucial role in the proliferation of ESCs. Members of the miR-302-367 cluster are highly expressed in early stages of embryonic development [59]. This miRNA cluster targets genes that are involved in epigenetic mechanisms. For example, the miRNA cluster downregulates lysine demethylases and CpG binding proteins MECP1-p66 and MECP2 [59]. This facilitates the transcription of pluripotent genes and thereby contributes to the sustenance of pluripotency in mammalian ESCs [59]. Furthermore, it has been demonstrated that the promoter of miR-302-367 is activated when bound by *OCT4, SOX2*, which are core transcription factors directly involved in the maintenance of ESCs [59, 60]. It has been also shown that this cluster promotes pluripotency in ESCs by targeting the SMAD signaling pathway and the PI3K/PKB signaling molecules. MiR-302 inhibits the expression of transforming growth factor beta-receptor 2 (*TGFBR2*) and *RAS* homolog gene family member C (*RHOC*), which leads to a reduction of epithelial-mesenchymal transition [59, 61, 62]. In addition, the miR-302 cluster has suggested to negatively regulates p21 and *LATS2* activity in both hESCs and mESCs [63, 64]. These molecular mechanisms enlighten the important role of the miR-302-367 cluster with respect to pluripotency and cell cycle modulations.

Another well-known miRNA family involved in the regulation of cell cycle progression is the let-7 family, which consist of let-7a-1, a-2, a-3, b, c, d, e, f-1, f-2, g, i and miR-98. Members of this miRNA family affect the G1/S transition of ESCs differently than the above-described ESCC miRNAs. While most of the ESCC miRNAs are related to promote self-renewal, the let-7 miRNAs suppress self-renewal [35, 52]. The mechanism underlying this antagonistic effect remains unclear. However, it has been suggested that the ESCC miRNAs positively regulate the expression of *LIN28*, which through a negative feedback loop suppress the let-7 maturation [65, 66].

Two other miRNAs known to affect the regulation of ESCs are miR-195 and miR-372a. Both miRNAs are highly enriched in hESCs compared to differentiated cells and their function also relies on maintaining the proliferative capacity of hESCs [67]. For example, ectopic expression of miR-195 results in reduced expression of the G2/M cell cycle checkpoint kinase *WEE1* and an enhancement of BrdU incorporation [67, 68]. Ectopic expression of miR-372 has also shown to reduce the p21 expression levels in Dicer-knockdown hESCs [67].

Human ESCs have the therapeutic potential to treat a myriad of disorders by cell replacement. In theory, ESCs could be used in regenerative medicine, drugs discovery and disease modeling. However, the usage of ESCs as clinical application is limited because of high tumorigenicity and ethical restrictions. A miRNA-based therapy that use induced pluripotent stem cells (iPSC) might overcome these limitations. In this regard, ectopic expression of ESCC miRNAs may contribute to expansion of stem cells for regenerative medicine purposes [12, 20, 44].

### Somatic Stem Cells

An extensive body of research has revealed the role of miRNAs in the cell cycle regulation of somatic stem cells [45, 69, 70]. In particular, studies with tissue specific Dicer-knockout or Dgcr8-deficient mice have demonstrated that miRNAs are essential regulators of proliferation, survival and differentiation in somatic stem cells [71]. In the following paragraphs, the role of miRNAs in the cell cycle regulation of hematopoietic and mesenchymal stem cells will be discussed. The associations of miRNAs with other somatic stem cells are summarized in Table 1.

**Table 1:** miRNAs associated with cell cycle regulation in somatic stem cells

Stem cell	miRNA ID	Potential target gene(s)	Reference
Epidermal	miR-205	PI3K-AKT	[72]
	miR-203	*SNAI2*, p63, *SNAP2*	[73]
	miR-34	p63	[74]
	miR-184	*NOTCH*, p63, *FIH1*	[75]
	miR-214	*WNT*/*β-catenin*	[76]
Neural	miR-9	*TLX, BAF53A*	[77]
	miR-137	*TLX*	[78]
	miR-184	*MBD1*	[79]
	miR-195	*MBD1*	[80]
	miR-124	*SOX-2, PTBP1, SCP1*	[81–83]
	miR-302	*p53, OCT4, SOX2, NANOG*	[84]
	miR-148b	*WNT*/*β-catenin*	[85]
	miR-138	*TRIP6*	[86]
Muscle	miR-27	*PAX3*	[87]
	miR-322	*CDC25A*	[88]
	miR-206	*HDAC4, PAX7*	[89, 90]
	miR-1	*HDAC4, PAX7*	[90]
	miR-133	*SRF, MALAT1*	[91]
	miR-221	*PI3K-AKT*	[92]
	miR-143	*IGFBP5, ERK1*/*2*	[93]
	miR-486	*PAX7*	[94]

**Fig. 2:**
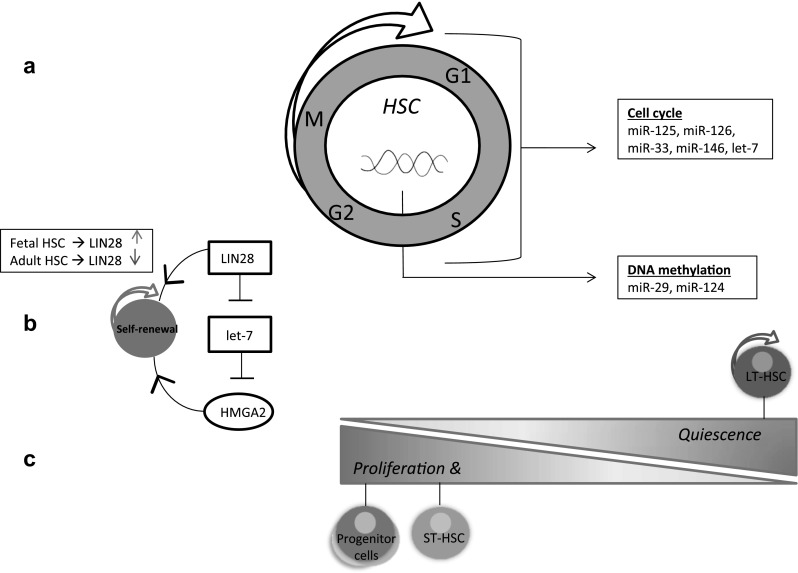
**miRNA-mediated regulation of cell cycle in HSCs**. (**a**) The schematic describes miRNAs (e.g. miR-125, miR-126, miR-33, miR-146 and let-7) with critical roles in the cell cycle regulation in adult HSCs by directly targeting cell cycle components. Furthermore, miR-29 and miR-124, which target components involved in DNA methylation, indirectly influence the expression of cell cycle-associated genes. **(b)** The LIN28-HMGA2 feed-forward loop is among the most important mechanisms that drive fetal HSC self-renewal. *LIN28* is highly expressed in fetal HSCs compared to adult HSCs. As *LIN28* directly inhibits let-7 expression, this indicates the important role of miRNA let-7 upon stem cell differentiation. Decreased level of let-7 has resulted in higher expression of *HMGA2*, which induces self-renewal. Additionally, *LIN28* can acts independently of the let-7 family and contributes to self-renewal [95, 96]. **(c)** Adult HSCs are a heterogeneous population that differ in self-renewal and differentiation capacity based on their surface markers. Long-term HSCs (LT-HSCs) are predominantly quiescent (c-kit^+^ Sca-1^+^ Lin^−^ Flk-2^−^ CD34^−^) [103]. However, a large fraction of short term-HSCs (c-kit^+^ Sca-1^+^ Lin^−^ Flk-2^−^ CD34^+^) gives rise to the differentiated progeny, and also shows greater cell proliferation capacity than LT-HSCs [102, 103]. Progenitor cells are associated with proliferation and differentiation into hematopoietic lineages. KSL (c-kit^+^ Sca-1^+^ Lin^−^) with high CD150^+^ expression may give predominant rise to myeloid linages, whereas KSL-CD150^−^ are more likely to a lymphoid outcome [104]. Several studies also demonstrate that specific miRNAs are differentially expressed among HSCs and progenitor cells

Hematopoietic stem cell (HSC) development has been characterized by several mechanisms that lead to generating multiple cell lineages. Adult HSCs are predominantly quiescent (in the G0 phase) compared to fetal HSCs [4]. Well established is the self-renewal function of the *LIN28* gene, which is highly expressed in fetal HSCs compared to adult HSCs (Fig. 2b) [95, 96]. This is a form-feedback loop which includes the downregulation of let-7 through *LIN28*, and subsequently downregulation of *HMGA2*. Given that *HMGA2* enhances the self-renewal capacity, the LIN28-HMGA2 pathway is crucial in stem cell development [97]. Most of the previous research has focused on determining the expression of miRNAs in hematopoietic stem and progenitor cells during lineage differentiation [98]. Several studies have also reported differential miRNA expressions between HSCs, hematopoietic progenitor cells and both myeloid and lymphoid linages (e.g. T cell, B cell, Granulocyte, Monocyte, Erythrocyte), demonstrating that miRNAs are involved in the differentiation of specific hematopoietic lineages [95, 99–101]. Although the conventional model suggests that hematopoietic lineages are derived from a common HSC, more recent research revealed that a rather large number of progenitor cells are the main drivers behind steady-state hematopoiesis and clonal diversity [102]. In this regards, short-term HSCs could support the heterogeneous range of progeny [102]. Taken the functional role of miRNAs into consideration, both progenitor cells and diverse miRNAs may be equally important for clonal expansion and hematopoiesis.

For example, miRNAs are differentially expressed between long term hematopoietic stem cells (LT-HSCs) and short term HSCs, which are defined by a combination of cell surface markers such as c-Kit^+^/Sca-1^+^/Lin^−^ (KSL). Based on the expression levels of cell surface markers including CD34, Flk-2, CD150, CD48, CD224, c-Kit, Sca-1, and Lin, the heterogeneous population of HSCs differ in proliferation and differentiation capacity [104]. The transition of HSCs into progenitor cells is related with a switch from quiescent into rapid proliferating cells, and subsequently an alteration in expression of surface makers (Fig. 2c). Therefore, the expression of cell cycle related miRNAs in exclusively progenitor cells is likely to be involved in the alteration of cell cycle duration [70]. One of the enhanced expressed miRNAs in LT-HSCs is the miR-125 cluster (miR-125a, miR-125b1, miR-125b2). The expression of miR-125 has been shown to be associated with self-renewal and expansion of the stem cell population in vivo [105–107]. Furthermore, miR-29a has been revealed to regulate the G1/S transition in hematopoietic progenitor stem cells. MiR-29a promotes the self-renewal capacity by targeting a subset of genes that are involved in cell cycle progression, including *CDC42EP2* and *HBP1* [108]. Recently, Lechman et al. demonstrated that miR-126 can control the cell cycle progression by targeting the PI3K/AKT/MTOR pathway [109]. They showed that overexpression of miR-126 results in an increased percentage of quiescent cells, whereas a knockdown of miR-126 lead to enhanced proliferation and differentiation of HSCs [109–111].

Additionally, previous studies have suggested miR-125 and miR-126 as potential target treatment for acute myeloid leukemia (AML) [112, 113]. An indication for the potential therapeutic function is based on the alternated expression of these miRNAs between CD34^+^ CD38^−^ HSC and CD34^+^ CD38^−^ leukemic stem cells. A reduction of miR-126 stimulates the PI3K/AKT/MTOR pathway in HSCs and will result in an increased number of HSCs, while this effect decreases the self-renewal capacity in CD34^+^ CD38^−^ leukemic stem cells [112]. Although this miRNA-based treatment holds promising capacity to in vivo experiments, issues with respect to toxicity and delivery need to be solved before application in AML patients [112].

Mesenchymal stem cells (MSCs) are multipotent cells that originate from bone marrow stroma, but are present in various tissues such as adipose tissue, bone, skeletal muscle, cartilage and tendon [114]. Evidence suggests that miRNAs are closely involved in the regulation of MSC differentiation into specific cell lineages [101, 115–117]. The role of miRNAs in proliferation and cell cycle regulation of human MSCs has been investigated through Drosha and Dicer knockdown studies [118]. These studies have shown a significant increase in the number of cells in G1 phase and a reduced proliferation rate of MSCs [118]. In the same study, Drosha knockdown in MSCs resulted in a decrease of pRb and an increase in p16 and p15 levels [118]. Other studies have been implicated miR-16 and miR-143 in the regulation of MSC proliferation and differentiation. In this regard, miR-16 has been shown to inhibit MSC proliferation and induce cell cycle arrest by targeting cyclin E [119]. Likewise, miR-143 targets *ERK5* (member of MAPK family), which itself decreases the expression of cyclin D and CDK6. This reduces cell entry into S phase, suggesting miR-143 to be a negative regulator of the cell cycle progression [120, 121]. Moreover, a number of miRNAs have determined to control the differentiation into specific linages, such as osteoblasts [122]. For example, Peng et al. demonstrated that miRNAs promote the osteogenic differentiation of MSCs via *BMP, WNT*/*β-catenin* and *NOTCH* signaling pathways. Among them, miR-27 promotes differentiation by targeting *APC*, which modulates the G2/M transition [122, 123]. On the other hand, miR-27 expression is shown to be downregulated upon adipocyte differentiation [124, 125]. Several cell cycle associated genes, including *ERK1*/*2, ERK5, TGF-β1* and *KLF5* are related to adipocyte differentiation, which is explained by miRNA regulation [126]. Notably, miR-143, miR-448 and miR-375 have been reported as negative regulators and miR-21 as positive regulator of adipocyte differentiation [126].

### Cancer Stem Cells (CSCs)

Altered expression and molecular abnormalities of the cell-cycle-regulatory proteins, such as pRB, p53, CDKs, CDKIs and cyclins, play a central role in cancer initiation and progression [17, 127–129]. Notably, it has been suggested that a class of cancer cells with characteristics of stem cells, so-called cancer stem cells (CSCs), are responsible for tumor initiation, invasion, metastasis and chemoresistance [130, 131]. As discussed previously in this review, miRNAs have the ability to suppress apoptosis and promote proliferation by interplaying with the cell cycle components. Therefore, miRNAs and CSCs share common properties with respect to tumorigenesis. The transcriptional levels of several miRNAs have shown to vary between normal stem cells and CSCs [132]. Furthermore, associations between either cell cycle components including cyclins and transcription factors or miRNA expression and specific CSC markers have been investigated [133, 134]. Hence, miRNAs as regulators of CSCs have gain attention in recent years in multiple fields of research [131, 133, 135, 136]. The associations between miRNAs expression and various cancers are summarized in Table 2. In the following paragraph, some of the main CSC-related miRNAs will be discussed.

**Table 2:** miRNAs associated with the cell cycle progression in cancer stem cells

Cancer type	miRNA ID	Potential target gene(s)	Exp. of miRNA	Reported biological effect	Reference
Breast	let-7	*LIN28*	Downregulated	Upregulation of *LIN28* results in supporting *RAS, MYC* and *HMGA2*	[137]
	miR-21	*PTEN*	Upregulated	Promote PI3K/AKT signaling activation through directly inhibiting *PTEN* expression	[138]
	miR-221/222	*PTEN*	Upregulated	Promote AKT/NF-κβ/COX-2 pathway by targeting *PTEN*	[139]
	miR-93	*JAK1, SOX4, STAT3, AKT, EZH1, HMGA2*	Upregulated	Regulate CSC proliferation	[140]
	miR-34	*CDK4, CDK6, NOTCH1*	Downregulated	Regulate p53	[141]
	miR-16	*BMI1*	Upregulated	Inhibit DNA repair by repressing *BMI1*	[142]
	miR-200	*ZEB1, ZEB2, WNT-signaling*	Downregulated	Reduction of EMT	[143]
	miR-494-3p	*PAK1*	Downregulated	Inhibit proliferation via MAPK by targeting *PAK1*	[144]
Liver (HCC)	miR-34	*Cyclin D1, BCL2*	Downregulated	Regulate p53	[145]
	miR-365	*BCL2*	Upregulated	Apoptosis	[146]
	miR-31	*HDCA2, CDK2*	Downregulated	Induction of p16 and p21. Repression of cyclin D, CDK4, CDK2	[147]
	miR-26a	*EZH2*	Upregulated	Reduction of EMT	[148]
	miR-150	*GAB1*	Downregulated	Suppress proliferation and invasion via MAPK pathway by targeting *GAB1* and *ERK1*/*2*	[149]
Head and Neck	let-7	*ABCB1*	Downregulated	Reduction of cell proliferation	[150]
Pancreatic	let-7	*LIN28*	Downregulated	Inhibit EMT, induces cell cycle arrest when *LIN28* is reduced	[151]
	miR-21	*PTEN, PDCD4*	Upregulated	Promote metastasis	[152]
	miR-203	*ZEB1, ZEB2*	Downregulated	Reduction of EMT	[153]
	miR-34	*BCL2, NOTCH1*/*2*	Downregulated	Regulate p53	[136]
	miR-17-92	*p21, p57, TBX3*	Downregulated	Maintain stemness characteristics in pancreatic CSC. Downregulation of *MYC*	[154]
Prostate	let-7	*LIN28*	Upregulated	Upregulating cell cycle via cyclin D1	[155]
	miR-100	*CDK6, RB1, mTOR*	Downregulated	Regulation of cell growth	[156]
	miR-34	*Cyclin D1, CDK4, CDK6, c-MET, CD44*	Downregulated	Mediating p53. Tumor metastasis	[157]
	miR-221/222	*p27*/*Kip1*	Upregulated	Regulate activation of cyclin E and cyclin D	[158]
Glioblastoma	miR-124	*CDK6*	Upregulated	Inhibit cell proliferation	[159]
	miR-137	*CDK6*	Upregulated	Inhibit cell proliferation	[160]
	miR-128	*BMI1*	Upregulated	Decreasing cell proliferation in *IDH1* mutant glioma	[161]
	miR-23b	*HMGA2*	Upregulated	Cell cycle arrest and proliferation inhibition	[162]
	miR-125b	*CDK6, E2F3, CDC25A*	Downregulated	Induce G1/S cell cycle arrest	[163]
	miR-34	*BCL2, NOTCH1*	Downregulated	Targeting p53. Anti-apoptotic, increase cell proliferation	[164]
Lung	miR-605	*LATS2*	Upregulated	Promote cell proliferation, migration and invasion	[165]
	let-7	*KRAS, MYC, CDK6 HMGA2, TGFBR2*	Downregulated	Suppression of multiple oncogenic members	[166]
	miR-21	*MDM4*	Upregulated	Repress *MDM4* to activate p53	[167]
	miR-15a/ miR-16	*RB*	Downregulated	Cell cycle arrest	[168]

The miR-17-92 cluster affects the cell cycle by targeting *E2F-1* and cyclin D as well as it cooperates with the oncogene *MYC* to prevent apoptosis in CSCs [169–172]. Li et al. investigated the miR-17-92 target genes involved in the *MYC* suppression. They demonstrated that the functionalities of the miR-17-92 target genes rely on multiple DNA replication, cell cycle regulation, chromosome organization, RNA transcription or protein metabolism [51]. Similarly, this miRNA cluster is shown to coordinate the timing of cell cycle progression by modulating expression of *BMI1, PTEN, RBL2* and p21 [154, 173–176].

Other important regulators of CSCs are the members of the let-7 family. Evidence suggests that let-7 is among the most important miRNAs involved in tumor progression and chemoresistance [131, 177]. The expression of the let-7 family is reduced in various types of tumor cells, including breast, head and neck squamous (HNSCC), lung, pancreatic, neuroblastoma cells, among others [131, 133, 178, 179]. Accordingly, decreased expression of let-7 has resulted in overexpression of oncogenes *MYC, RAS, HMGA2* and *BLIMP1* [115, 177, 180]. Furthermore, members of the let-7 family have been recognized as negative regulators of *PTEN* that inactivate the PI3K/AKT/MTOR pathway. The let-7 family has also shown to be involved in suppressing the epithelial-to-mesenchymal transition (EMT), which is related to metastasis and chemoresistance and therefore a characteristic of CSCs [131, 177]. Multiple genes involved in cell cycle progression are suggested to be targets for the let-7 family. The latter include cyclin D, cyclin A, *CDK1, CDK2, CDK4, CDK6, CDK8* and *CDC25A* [115, 177, 180]. Also, it has been shown that the RNA binding protein *LIN28* inhibits let-7 by stimulating cellular proliferation via cyclin D, *CDK2* and *CDC25A* and thereby contribute to the maintenance of stemness characteristics of CSCs [46, 181]. *LIN28* has been recognized as an oncogene, as it promotes tumor progression by repressing let-7 [177]. Previous studies based on let-7 expression and tumor progression display that ectopic expression of let-7 was sufficient enough to inhibit proliferation and clonal expansion in vitro and tumor recurrence in prostate cancer cells in vivo [173].

The next miRNA family, consisting of miR-34a, b, and c, is well-studied regarding to cell cycle progression and its expression is downregulated in several types of cancer cells including lung adenocarcinomas, colon cancer and liver cancer (HCC) [141, 167, 182–185]. MiR-34a induces both G1/S cell cycle arrest and cell senescence [167]. Reduced expression of miR-34 has been associated with enhanced levels of *BCL2* and *NOTCH*, which are target genes for tumor suppressor gene p53 [131, 135, 167]. Similarity, miR-34 promotes apoptosis via *Caspase 3*, and therefore increases sensitivity for anti-cancer treatment [135]. By regulating CDK6, cyclin D1 and *E2F*, miR-34 negatively affects cell cycle progression in colon cancer cells [131, 184, 185]. In addition, miR-34 represses pluripotency genes inclusive of *NANOG, SOX2* and *MYC* [135]. Thus, overexpression of this miRNA family may cause an accumulated percentage of cells in the G0/G1 phase and significantly reduces the population of cells in the S phase.

MiR-31 has also shown to be inversely correlated with metastasis, since its high expression in liver cancer is linked with a poor prognosis in patients. Kim et al. showed that ectopic expression of miR-31 evokes an overexpression of CDK2 and *HDAC2* [147]. They demonstrated that through abnormal expression of *HDAC2*, negative cell cycle regulators p16/INK4A, p19/INK4D and p21/Cip1 are induced.

Furthermore, an oncogenic role has been reported for the miR-15a/16 family in chronic lymphocytic leukemia (CLL), pituitary adenomas, and gastric cancer [186, 187]. On the other hand, this miRNA family is shown to act as a tumor suppressor in a subset of B cell lymphoma, where deletion of this miRNA family in a subset of B cell lymphomas resulted in chronic lymphocytic leukemia in mice [188]. In fact, miR-15a and miR-16 display an anti-proliferative potential in this type of cancer stem cell by silencing *BCL2* and activating the intrinsic apoptosis pathway [189, 190]. In addition, some studies revealed the miR-15a/16 family as regulator of various cyclins, including cyclins D1 and D2 and cyclin E1, and pRb [168, 180, 191].

An additional miRNA that has been suggested as an oncomiR, through targeting multiple signaling pathways, is miR-21 [33]. Upregulation of miR-21 has an oncogenic potential in a wide range of tumors including lung, breast, pancreatic, brain and colon cancers, through downregulation of p21 and tumor suppressor genes *PTEN* and *PDCD4* [33, 192–194]. MiR-26a is also suggested as a negative regulator of cancer cell proliferation by targeting cyclins D2 and E2, and CDK6. It has been established that overexpression of miR-26a results in cell cycle arrest in human liver cancer cells in vitro [195, 196].

## Concluding Remarks and Future Prospects

A growing body of evidence has addressed the potential role of miRNAs in cell cycle regulation of stem cells. In light of recent discoveries about the role miRNAs in self-renewal, proliferation and differentiation, it is crucial to unravel the complex mechanisms and molecular interactions within this field of research. In this review, we outlined the most established miRNAs involved in the cell cycle progression of stem cells. We highlighted several clusters and single miRNAs that may control self-renewal and maintenance of the pluripotency status in ESCs. These include but are not limited to ESCC miRNAs (miR-290-295, miR-302, miR-17-92, miR-106b-25 and miR-106a-363), which are functionally upregulated to suppress negative regulators and to enhance pluripotent transcription factors such as *NANOG* and *MYC* in an epigenetic manner [45].

Furthermore, specific profiles of miRNA expression in distinct somatic stem cell lineages are linked with developmental control by keeping several multipotent stem cells (e.g. HSCs) in a quiescent state. Previous research based on Dicer-knockout and Dgcr8-deficient mice have elucidated that miRNAs are expressed temporally and spatially among somatic stem cells and precursor cells [37]. It is crucial for somatic stem cells like HSCs to keep a balance between quiescent state and proliferating state. To accomplish that, a complex network of miRNAs exists that inhibit positive cell cycle regulators such as cyclins, as well as miRNAs modulating anti-apoptotic properties. Complex interactions between miRNAs, transcription factors and cell cycle-mediated components may control the gene expression upon differentiation of multipotent stem cells into progenitor cells and mature cells.

It is clear that abnormalities in the cell cycle are related to tumorigenesis and previous studies have highlighted the significant importance of miRNAs in the regulation of CSCs [132]. Since CSC features are linked to metastasis, invasion and therapeutic resistance, it is of main clinical relevance to unravel the interactive properties between CSC-related miRNAs and cell cycle components. From the data available so far it appears that there is a great overlapping role between ESCC miRNAs that are expressed in both ESCs and CSCs. However, a subset of miRNAs is characterized as tumor suppressor genes as they are expressed regarding anti-proliferating features by targeting oncogenic pathways including *MYC*. Those miRNAs, including let-7, miR-34, miR-31 and miR-17-92 family, are of major interest since they are associated with a good prognosis in cancer patients. Future research should focus on targeting the CSC-related miRNAs involved in oncogenic pathways since they will provide a more effective approach to exterminate CSCs. Subsequently, a miRNA based method for cancer treatment is highly target driven as it interferes with specific abnormalities in the cell cycle within the tumor microenvironment.

Collectively, this review marks several noteworthy insights into the cell cycle regulation of stem cells by miRNAs. Understanding the tightly regulated molecular networks in which miRNAs are interacting, will greatly enhance our knowledge in the development of both healthy and disease states of the human body.
